# Spatial transcriptome analysis reveals de novo regeneration of poplar roots

**DOI:** 10.1093/hr/uhae237

**Published:** 2024-08-20

**Authors:** Kaiwen Lv, Naixu Liu, Yani Niu, Xiehai Song, Yongqi Liu, Zhiliang Yue, Muhammad Ali, Qiuyue Guo, Chunyu Lv, Dongdong Lu, Shaoman Zhang, Yangyan Zhou, Bosheng Li

**Affiliations:** Shandong Provincial Key Laboratory of Precision Molecular Crop Design and Breeding, Peking University Institute of Advanced Agricultural Sciences, Shandong Laboratory of Advanced Agricultural Sciences in Weifang, Shandong 261325, China; Shandong Provincial Key Laboratory of Precision Molecular Crop Design and Breeding, Peking University Institute of Advanced Agricultural Sciences, Shandong Laboratory of Advanced Agricultural Sciences in Weifang, Shandong 261325, China; Shandong Provincial Key Laboratory of Precision Molecular Crop Design and Breeding, Peking University Institute of Advanced Agricultural Sciences, Shandong Laboratory of Advanced Agricultural Sciences in Weifang, Shandong 261325, China; Shandong Provincial Key Laboratory of Precision Molecular Crop Design and Breeding, Peking University Institute of Advanced Agricultural Sciences, Shandong Laboratory of Advanced Agricultural Sciences in Weifang, Shandong 261325, China; Shandong Provincial Key Laboratory of Precision Molecular Crop Design and Breeding, Peking University Institute of Advanced Agricultural Sciences, Shandong Laboratory of Advanced Agricultural Sciences in Weifang, Shandong 261325, China; Shandong Provincial Key Laboratory of Precision Molecular Crop Design and Breeding, Peking University Institute of Advanced Agricultural Sciences, Shandong Laboratory of Advanced Agricultural Sciences in Weifang, Shandong 261325, China; Shandong Provincial Key Laboratory of Precision Molecular Crop Design and Breeding, Peking University Institute of Advanced Agricultural Sciences, Shandong Laboratory of Advanced Agricultural Sciences in Weifang, Shandong 261325, China; Shandong Provincial Key Laboratory of Precision Molecular Crop Design and Breeding, Peking University Institute of Advanced Agricultural Sciences, Shandong Laboratory of Advanced Agricultural Sciences in Weifang, Shandong 261325, China; Shandong Provincial Key Laboratory of Precision Molecular Crop Design and Breeding, Peking University Institute of Advanced Agricultural Sciences, Shandong Laboratory of Advanced Agricultural Sciences in Weifang, Shandong 261325, China; Shandong Provincial Key Laboratory of Precision Molecular Crop Design and Breeding, Peking University Institute of Advanced Agricultural Sciences, Shandong Laboratory of Advanced Agricultural Sciences in Weifang, Shandong 261325, China; Shandong Provincial Key Laboratory of Precision Molecular Crop Design and Breeding, Peking University Institute of Advanced Agricultural Sciences, Shandong Laboratory of Advanced Agricultural Sciences in Weifang, Shandong 261325, China; Salver Academy of Botany, RiZhao, Shandong 262300, China; Shandong Provincial Key Laboratory of Precision Molecular Crop Design and Breeding, Peking University Institute of Advanced Agricultural Sciences, Shandong Laboratory of Advanced Agricultural Sciences in Weifang, Shandong 261325, China

## Abstract

Propagation through cuttings is a well-established and effective technique for plant multiplication. This study explores the regeneration of poplar roots using spatial transcriptomics to map a detailed developmental trajectory. Mapping of the time-series transcriptome data revealed notable alterations in gene expression during root development, particularly in the activation of cytokinin-responsive genes. Our analysis identified six distinct clusters during the second and third stages, each corresponding to specific anatomical regions with unique gene expression profiles. Auxin response *cis*-elements (AuxREs) were prevalent in the promoters of these cytokinin-responsive genes, indicating a regulatory interplay between auxin and cytokinin. Pseudo-temporal trajectory analysis mapped the differentiation from cambium cells to root primordium cells, revealing a complex pattern of cell differentiation. *SAC56* and *LOS1* emerged as potential novel biomarkers for enhancing root regeneration, with distinct spatial expression patterns confirmed by *in situ* hybridization. This comprehensive spatial analysis enhances our understanding of the molecular interactions driving root regeneration and provides insights for improving plant propagation techniques.

## Introduction

Plants possess the remarkable ability to regenerate new tissues and organs from somatic cells following injury. This regeneration begins when cells detect specific signals that trigger a series of events: dedifferentiation, proliferation, and ultimately, the adoption of new cellular roles. Such a dynamic response not only repairs damaged tissues but also facilitates the growth of new organs from the site of injury [1]. In both forestry [2] and horticultural practices [3] such as grafting and tissue culture, cutting techniques exploit this regenerative capability to propagate root organs from scratch, preserving specific plant characteristics while remaining easy to use and broadly applicable.

Upon injury, plant tissues such as stem segments or leaves perceive wound signals, triggering a rapid response from the plant hormone jasmonic acid (JA). This response is pivotal for initiating auxin biosynthesis, which promotes the formation of adventitious roots in explants [4, 5]. Auxin, quickly synthesized at the site of injury, is directed to these regions in high concentrations. This hormone guides the transformation of stem cells at the wound site into root primordia [6, 7]. Auxin directly stimulates the expression of *WUSCHEL RELATED HOMEOBOX 11* (*WOX11*) through signal transduction in the initial cells, which in turn activates *WOX5/7* and *LATERAL ORGAN BOUNDARIES DOMAIN16* (*LBD16*), which are characteristic molecular markers of root primordial cells [8–10].

As the root primordium develops, high auxin levels within the meristem impede patterning processes. To preserve the integrity of the stem cell niche, it is crucial to restrict auxin to the tip of the meristem. It is believed that locally synthesized cytokinin, which accumulates during meristem formation, helps balance auxin levels, thereby containing its spread within the stem cell niche [11–13]. Additionally, there is a noted gradual decrease in *LBD16* expression within the root meristem, while *WOX5/7* expression becomes localized to the stem cell niche [8, 12]. Furthermore, the proteins *PLETHORA 1* (*PLT1*) and *PLT2*, along with *SCARECROW* (*SCR*) and *SHORT ROOT* (*SHR*), play essential roles in defining the quiescent center and maintaining stem cell activity within the root apical meristem [14–16]. Despite the recognized importance of cytokinin in root development, comprehensive research on this topic remains limited.

Recent advancements in spatial and single-cell transcriptomics have revolutionized the study of gene expression in tissue and organ contexts. These technologies allow for precise gene localization within samples and have been instrumental in delineating cellular characteristics across developmental gradients in various plant species [17–19]. Notably, the use of 10× Visium technology has provided new insights into early floral development in *Phalaenopsis* Big Chili [20]. It has also enabled detailed analyses that reveal dynamic molecular maps of cambium differentiation during both primary and secondary growth of trees [21]. Additionally, the combination of high-resolution anatomical analysis with spatial transcriptomics has allowed for a detailed characterization of cellular features in poplar stems, from primary to secondary vascular tissues [22]. This integrated approach, combining spatial transcriptomics with anatomical analysis, allows us to map gene expression with greater precision, offering a comprehensive view of cellular dynamics during root regeneration. In this study, we employ spatial transcriptomics to map the expression profiles of distinct cell types during the regeneration of poplar adventitious roots, focusing particularly on cytokinin-responsive genes within the root primordium and adventitious root. Our aim is to understand how cytokinin affects de novo root regeneration and its interaction with auxin in regulating this process. Through this comprehensive analysis, we seek to identify novel marker genes associated with root regeneration and elucidate the molecular mechanisms and signaling pathways involved. This research aims to provide new insights into the coordinated hormonal regulation of root regeneration, enhancing our understanding of plant developmental biology and informing strategies for improving plant propagation and growth.

**Figure 1:**
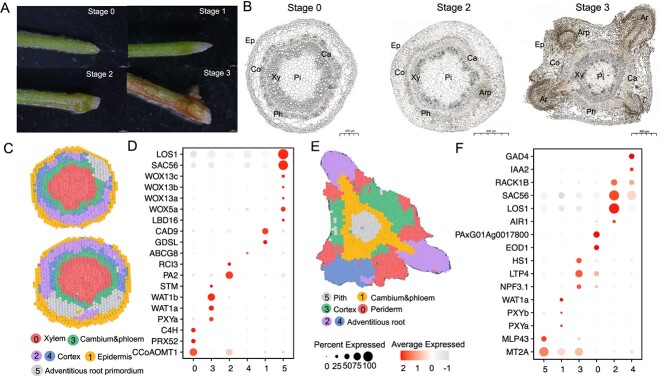
Identification of cell clusters during adventitious root regeneration. **A** Four stages of adventitious root regeneration. Photographic representation outlining the sequential stages in the development of adventitious roots, starting from stem segment detachment to the emergence of mature roots. **B** Paraffin sections displaying different cell types. Microscopic images of paraffin-embedded sections showing the diversity of cell types at various stages of root development. **C** Spatial distribution of clustering in Stage 2. Spatial transcriptome spots are divided into six distinct clusters, illustrating the spatial gene expression patterns in the second stage of root development. **D** Dot plot of marker gene expression in Stage 2. A dot plot showing the expression patterns of specific marker genes during the second stage, highlighting the activity within different clusters. **E** Spatial distribution of clustering in Stage 3. Similar to panel C, but showing the spatial transcriptome spots for the third stage of root development, again divided into six clusters. **F** Dot plot of marker gene expression in Stage 3. A dot plot similar to panel D, but detailing the expression of marker genes during the third stage of root development.

## Results

### Time-series analysis of gene expression during poplar adventitious root regeneration

To elucidate the molecular mechanisms driving the adventitious root regeneration in poplars, we adopted a time-series analytical approach. This method allowed us to capture the dynamic progression of adventitious root formation in poplar (Fig. S1, see online supplementary material). We conducted bulk RNA sequencing throughout this regeneration process, providing a comprehensive view of the genetic activities involved. The development of adventitious roots occurs in distinct, sequential stages (Fig. 1A). Stage 0: Detachment of stem segments from mature plants, sensing wound signals and other environmental cues [12], initiating the regenerative response. During this stage, the wound created by detachment serves as an important initiating factor for regeneration. Jasmonate rapidly accumulates in the wounded area and activates the expression of specific transcription factors, which in turn activate the auxin synthesis pathway, promoting adventitious root formation [4]. Stage 1: Polar transport of auxin to stem cells near the wound site. Auxin, the core hormone of root regeneration, drives the change in stem cell fate and sets the foundation for new root development [6, 7, 12]. Stage 2: The root primordium is formed at the base of the stem. During this phase, cells near the wound proliferate and differentiate into root primordium cells. These cells express key genes associated with root initiation, such as *WOX5* and *LBD16*, which direct the formation of adventitious root primordium cells [8, 9]. Stage 3: The adventitious root tip emerges from the base of the stem segment, extending outward and forming mature adventitious roots [12]. During this stage, the root primordium elongates and differentiates into specialized tissues. This growth allows the roots to connect with the soil, enabling nutrient uptake essential for the plant’s growth [23]. Throughout these stages, we systematically observed and recorded the changes in cellular organization through detailed paraffin sectioning, as depicted in Fig. 1B, providing a comprehensive understanding of the molecular and cellular basis of adventitious root regeneration in poplars.

We conducted a comprehensive analysis of the transcriptome data, comparing samples from various developmental stages to those from the initial abscission of stem segments in mature plants (designated as the 0-day sample in our study, see Fig. S1, see online supplementary material), we successfully identified a set of differentially expressed genes (DEGs) using criteria of fold changes greater than 2 and adjusted *P*-values (padj) less than 0.05, as detailed in Table S1 (see online supplementary material). We further subjected these DEGs to a Gene Ontology (GO) enrichment analysis (Table S1, see online supplementary material). The resulting analysis highlighted several pivotal biological processes that are specifically associated with the third day of poplar adventitious root regeneration. Notably, among the top 30 enriched GO terms, we observed a significant enrichment of processes related to lateral root development (GO:0048527), post-embryonic root morphogenesis (GO:0010101), and lateral root morphogenesis (GO:0010102). These findings provide compelling evidence of the active emergence of root primordia during this critical developmental stage, further emphasizing the dynamic transcriptional changes that occur during this process.

### Spatial transcriptome analysis of poplar tissues undergoing root regeneration

To gain deeper insights into the molecular events underpinning adventitious root development, we conducted spatial transcriptome experiments focusing on the emergence and maturation stages of adventitious roots. Despite limited numbers of root primordia observed in Stage 1, where cutting samples were cultured in rooting medium for three days, we selected the second stage—samples cultured for four days—as our primary focus for spatial transcriptome sequencing. This stage presented a richer abundance of root primordia, facilitating more robust data collection and analysis.

Using BSTMatrix, we performed a detailed analysis of spatial transcriptome data, which allowed us to examine gene expression patterns across these developmental stages. At Level 7 during the second stage, our analysis detected 27 927 genes with a median of 1777 genes expressed per cell (Table S2, see online supplementary material). During the third stage, 27 913 genes were detected with median of 556 genes expressed per cell (Table S2, see online supplementary material), highlighting the dynamic shifts in gene expression as development progressed.

Through meticulous examination of the spatial transcriptomic data, we identified six distinct clusters in the second developmental stage, each corresponding to specific anatomical regions with unique gene expression profiles (Fig. 1C and D). For example, Cluster 0 was characterized predominantly by xylem cells, with key genes such as *CCoAOMT1* [24], *PRX52* [25], and *C4H* [26] marking this differentiation. In contrast, Cluster 3, encapsulating the cambium and phloem, featured marker genes like *PXYa*, *PXYb* [27], *WAT1a*, *WAT1b*, and *STM* [28]. The cortex was mainly represented in Clusters 2 and 4, while the epidermis was grouped into Cluster 1, marked by genes such as *GDSL* [29]. Cluster 5, identified as the adventitious root primordia cluster, included the marker genes such as *WOX5a* [30] and *LBD16* [9]. The list of genes enriched by using Seurat can be found in Table S3 (see online supplementary material).

**Figure 2:**
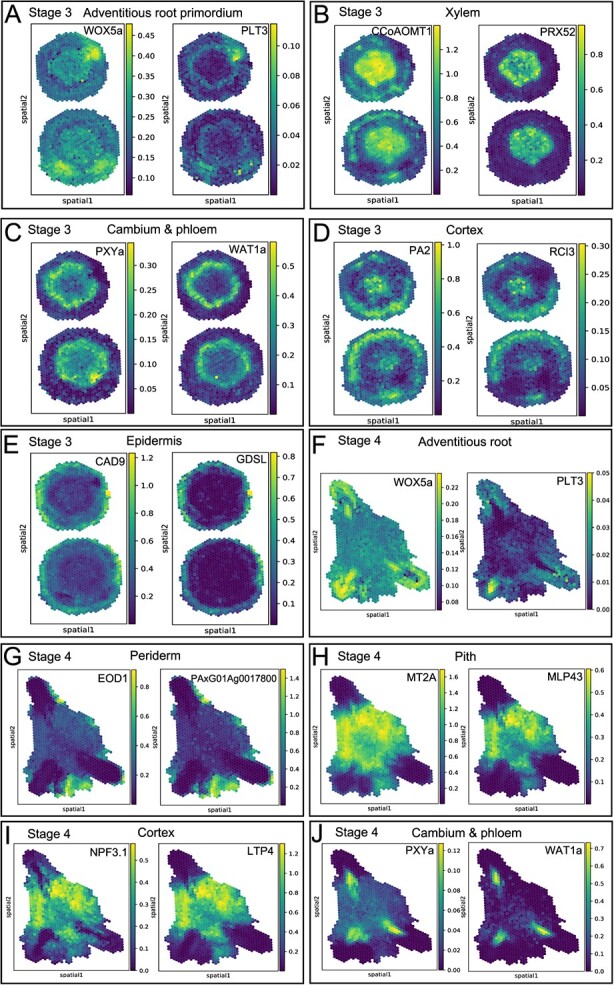
Spatial expression pattern of marker genes in adventitious root development. This figure includes a color gradient scale to represent gene expression levels. The scale ranges from dark blue, indicating low levels of expression, to yellow, signifying high levels of expression. This visual aids in identifying the intensity of gene activity across different regions within the samples.

Spatial transcriptomics has proven invaluable in obtaining a holistic view of gene expression within tissues, particularly when examining tissue-specific expression. By analysing the spatial expression patterns of marker genes such as *WOX5a* and *PLT3* [30], we observed a close match with the locations of adventitious root primordia (Fig. 2A). Expanding our analysis, we investigated the spatial expression of additional marker genes, which helped elucidate their roles in cellular and tissue functions, reflecting specific expression locations within the tissue structures (Fig. 2B–E).

The third developmental stage, detailed through six clusters (Fig. 1E and F), marked the full maturation of adventitious roots. Clusters 2 and 4 were particularly associated with mature adventitious roots (Fig. S2B, see online supplementary material), whereas Cluster 0 represented the periderm, and Cluster 5 the pith of the tree. Cluster 3 reflected the cortex, and Cluster 1 comprised the cambium and phloem. This stage allowed us to explore deeply the specific locations of marker gene expression, enhancing our understanding of the molecular dynamics during this critical phase of root development (Fig. 2F–J). This comprehensive spatial analysis not only confirmed the dynamic nature of root regeneration but also enhanced our understanding of the complex molecular interactions driving this process.

### Spatial mapping of hormone-responsive genes

Hormones play a pivotal role in orchestrating the complex processes of plant growth and development. To probe deeper into the molecular underpinnings of these processes, particularly in adventitious root regeneration, we analysed the expression of genes responsive to key hormones including auxin, cytokinin, abscisic acid, gibberellin, salicylic acid, and jasmonic acid. Using GO annotations for auxin (GO:0009733), cytokinin (GO:0009735), abscisic acid (GO:0009737), gibberellin (GO:0009739), salicylic acid (GO:0009751), and jasmonic acid (GO:0009753), we identified a comprehensive list of hormone-responsive genes detailed in Table S4 (see online supplementary material).

**Figure 3:**
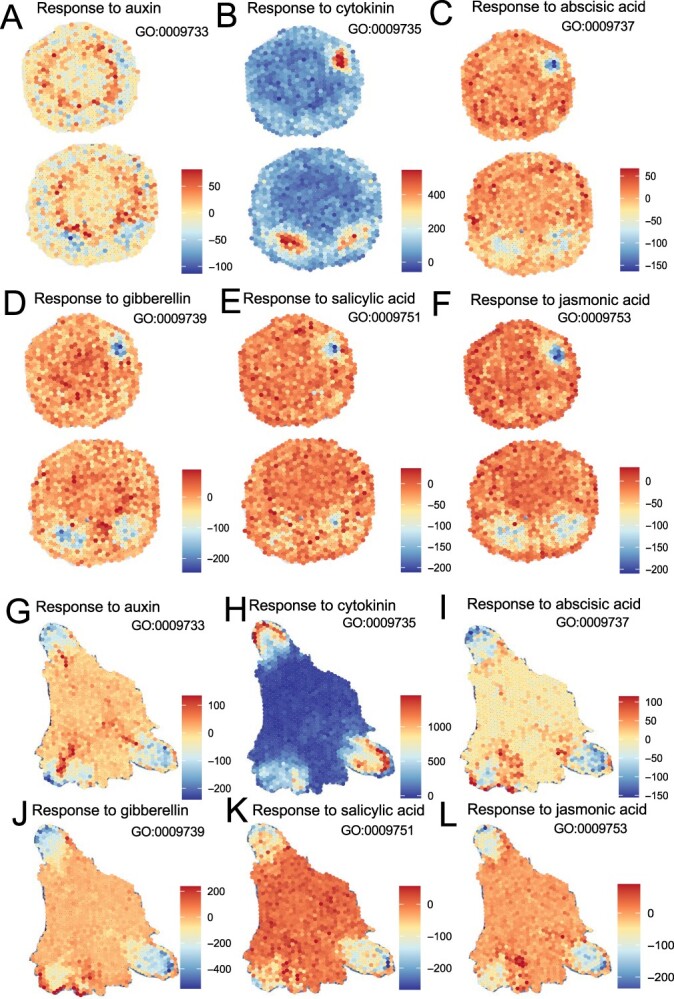
Spatial heatmap of hormone-responsive gene expression in adventitious root development. This heatmap visualizes the expression patterns of genes responsive to key hormones—auxin, cytokinin, abscisic acid, gibberellin, salicylic acid, and jasmonic acid—during the second and third stages of adventitious root development. The color gradient on the heatmap indicates gene expression levels in individual cells, with colors transitioning from blue (low expression) to red (high expression). This color coding helps delineate areas of intense hormonal activity and subtle responses across different cellular environments.

These genes were subsequently mapped to their spatial positions within the tissues undergoing root regeneration. As depicted in Fig. 3, a considerable expression of cytokinin-responsive genes was observed in the adventitious root primordium during the second stage of root development (Fig. 3B). This pattern persisted into the third stage, underscoring the crucial role of cytokinin in regulating the architecture of adventitious roots (Fig. 3H).

### Cytokinin’s role across developmental stages and genetic mechanisms underlying hormone sensitivity

The spatial analysis provides critical insights into the dynamic expression patterns of these hormone-responsive genes, elucidating their distinct roles throughout the stages of root development. Notably, our transcriptome data analysis revealed recurrent GO terms associated with cytokinin responses across all developmental stages, indicating its central role in root regeneration. Previous studies have highlighted that *ARF6/8* directly binds to auxin response *cis*-elements (AuxREs) on the promoters of key genes such as *RGI2* and *LBD16*, enhancing our understanding of hormone interplay in root development [31, 32]. Our findings further demonstrated that numerous cytokinin-responsive genes—291 out of 309 (94.17%)—contain AuxREs *cis*-elements in their promoters, suggesting a complex regulation by auxin signaling. Moreover, the transcription factor *WOX11* was found to promote the expression of *LBD16* by binding to WOXCEs on its promoter during the initiation of adventitious root primordium from detached leaves, reflecting the intricate genetic networks involved [10, 32]. Among the cytokinin-responsive genes analysed, 293 out of 309 (94.82%) were found to contain WOXCEs. Furthermore, 277 out of 309 (89.64%) of these genes contain both AuxREs and WOXCEs in their promoters, highlighting an overlap in the regulatory pathways governing root.

### Trajectory of adventitious root primordium differentiation from cambium

**Figure 4:**
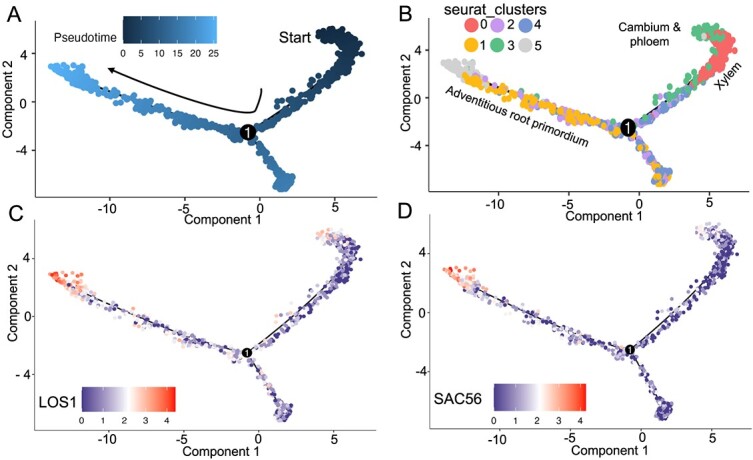
Trajectories of adventitious root regeneration. **A** and **B** The pseudo-time of the trajectory cell-type differentiation. This panel shows the trajectories of different cell clusters as they differentiate over time. Each dot represents a cell, plotted to demonstrate the progression from one cell type to another within the cambium-derived tissues. **C** and **D** Expression patterns of cytokinin-responsive genes during differentiation. These panels depict the expression responses of cytokinin-responsive genes along the differentiation trajectories. Each dot represents a cell, with the color indicating the level of gene expression as outlined in the color bar. The color gradient displays gene expression levels, where purple represents lower expression levels and red indicates higher expression levels.

To map the pseudo-temporal trajectory of the differentiation process in adventitious root primordia, we conducted an in-depth analysis of all cell clusters, constructing a trajectory that captures the dynamics of cell subtype evolution over time. As illustrated in Fig. 4A and B, starting from cambium cell (cluster 3), our observations revealed a complex pattern of cell differentiation. Some cells followed a trajectory leading to differentiation into xylem cell, while others transitioned into pholem, cortex, and epidermal cells, ultimately culminating in the formation of root primordial cells.

To connect the expression patterns of cytokinin-responsive genes with the differentiation pathways of root primordial cells, we focused on the roles of two specific genes: SUPPRESSOR OF ACTIN 56 (*SAC56*) and *LOS1*. The gene expression trajectories of these genes, as detailed in Fig. 4C and D, demonstrate a synchronized upregulation during the differentiation of root primordial cells. The gene expression trajectories of these genes, as detailed in Fig. 4C and D, demonstrate a synchronized upregulation during the differentiation of root primordial cells. Notably, *SAC56* and *LOS1* show connections to the cambium and phloem, suggesting that these tissues may serve as progenitors for AR development. This aligns with findings from previous studies [7, 33] which propose that cambium and phloem cells play pivotal roles in initiating root formation. This synchronous expression underlines the notable regulatory influence of cytokinin-responsive genes in directing the differentiation fate of root primordial cells.

### Identification of *SAC56*, *LOS1* as marker genes for adventitious root development

**Figure 5:**
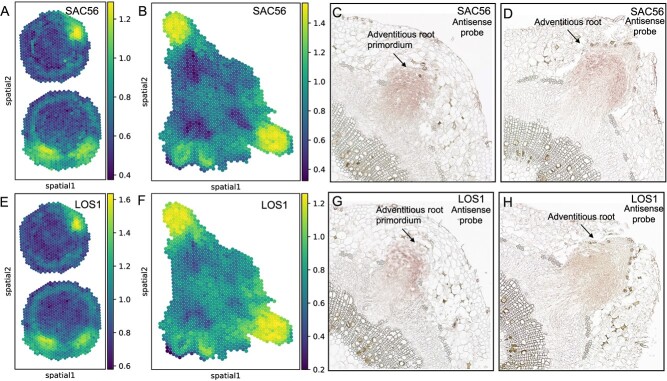
Identification of new marker genes for adventitious root regeneration. **A** and **B** Expression of *SAC56* during root development. These panels display the spatial transcriptome analysis results for *SAC56*, showing its expression in Stage 2 (Panel A) and Stage 3 (Panel B) of adventitious root regeneration. **C** and **D** Validation of *SAC56* expression. RNA in situ hybridization confirming the expression of *SAC56* in Stage 2 (Panel C) and Stage 3 (Panel D) of root development, supporting the transcriptome analysis findings. **E** and **F** Expression of *LOS1* during root development. Similar to panels A and B, these show the results of spatial transcriptome analysis for *LOS1*, detailing its expression in Stage 2 (Panel E) and Stage 3 (Panel F). **G** and **H** Validation of *LOS1* expression. RNA in situ hybridization confirming the expression of *LOS1* in Stage 2 (Panel G) and Stage 3 (Panel H), corroborating the transcriptome data.

Phosphoinositides (PIs), crucial signaling molecules in eukaryotes, play pivotal roles in plant growth and development. Specifically, SAC6-SAC8 PtdIns4P phosphatases are essential for auxin-mediated plant development [34]. LOS1, functioning as an elongation factor 2, induces dramatic changes in *Arabidopsis* root growth in response to auxin [35]. Through our spatial transcriptome data analysis, we observed that genes *SAC56* and *LOS1* demonstrate distinct spatial expression patterns during the second and third stages of adventitious root regeneration, primarily within the adventitious root primordium and the adventitious root (Fig. 5A, B, E, F). To further validate these observations, we performed *in situ* hybridization experiments using primers listed in Table S5 (see online supplementary material). The results from these experiments corroborate our spatial transcriptome findings, confirming the consistent expression patterns of these genes. Given their distinct expression patterns and crucial roles in root development, we designate *SAC56* and *LOS1* as marker genes for the adventitious root regeneration process in poplar.

To support the broader research community in accessing and analysing these gene expression patterns, we have uploaded our spatial expression data to our dedicated server (http://www.lilabpku-iaas.com:8678). Researchers can conveniently explore the detailed spatial expression patterns of these and other genes by referring to Fig. S3 (see online supplementary material), facilitating further studies and validation of our findings.

### Spatial distribution of auxin-related gene expression in cambium cells

**Figure 6:**
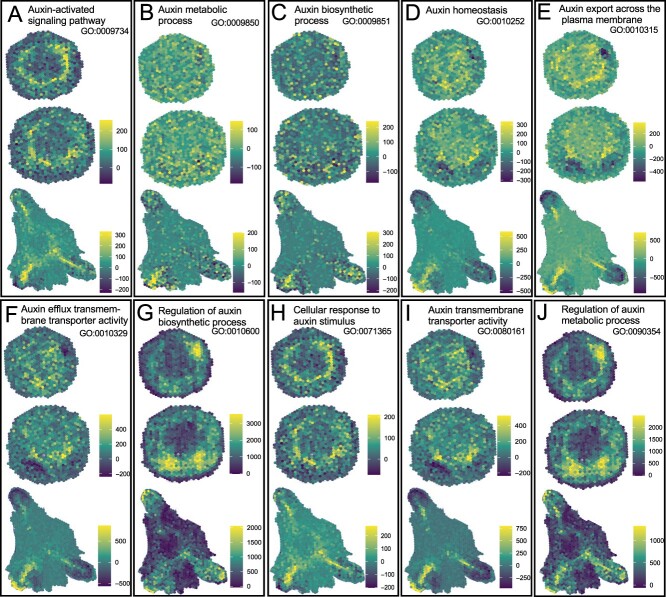
Spatial heatmap of auxin-related gene expression in adventitious root development. This heatmap illustrates the expression patterns of genes associated with auxin during the second stage of adventitious root development. The colors on the heatmap represent the levels of gene expression in individual cells. Dark blue indicates lower expression levels, while yellow indicates higher expression levels.

Understanding the spatial distribution of auxin-related gene expression is essential for elucidating the dynamics of adventitious root regeneration. A key question arises: In what specific areas within plant tissues do auxin-related genes become active during the process of root regeneration? To address this, we analysed GO enrichment across various developmental stages, as detailed in Table S1 (see online supplementary material). We identified 11 GO terms related to auxin expression, listed in Table S6 (see online supplementary material), and subsequently extracted the relevant genes. By mapping these genes to their specific tissue expression locations, we were able to examine the spatial expression patterns of auxin-related genes, as shown in Fig. 6. Our analysis revealed that a substantial number of genes associated with auxin expression are prominently expressed in the cambium cells. This finding highlights the critical role of cambium cells as sites of intense auxin-related genes, providing crucial insights into their specific functions during the complex process of adventitious root regeneration.

## Discussion

The development of adventitious root is an inherently dynamic process, particularly pronounced during the early stages that are crucial for determining cell fate. To capture this dynamic progression, our time-series analytical approach in bulk RNA sequencing captured the dynamic progression of adventitious root formation in poplar. The transcriptomic landscape revealed significant changes in gene expression associated with lateral root development, post-embryonic root morphogenesis, and lateral root morphogenesis during the third day of adventitious root regeneration (GO:0048527, GO:0010101, GO:0010102). These findings indicate the active emergence of root primordia during this stage, underscoring the importance of these developmental processes (Fig. 1A and B). Despite these insights, challenges arise from the uncertainty in pinpointing the exact rooting positions, complicating the acquisition of precise data through traditional frozen sectioning. To address these challenges, our spatial transcriptome experiments focused on the second stage of adventitious root development. This selection allowed us to capture essential insights during a crucial period of root primordia formation, overcoming limitations posed by the dynamic nature of these developmental processes. Our analysis, conducted at Level 7 with a cell resolution of 50 μm, highlighted the resolutions associated with various levels of spatial transcriptome data, as provided by Biomarker Technologies (Biomarker Technologies is a high technology enterprise that provides genomics sequencing services and single-cell spatial omics instrument equipment), though it is important to note the typical poplar cell size ranges from approximately 15–40 μm, potentially impacting the differentiation clarity between cell types (Table S7, see online supplementary material).

Following our spatial transcriptome experiments, pseudo-temporal trajectory analysis mapped the differentiation process from cambium cells to root primordial cells, revealing a complex pattern of cell differentiation into xylem, phloem, cortex, and epidermal cells, ultimately forming root primordial cells. This trajectory underscores the intricate molecular mechanisms driving cell fate determination during root regeneration (Fig. 4). This analysis underscored the intricate molecular mechanisms driving cell fate determination during root regeneration. In this context, we identified *SAC56* and *LOS1* as potential novel biomarkers for enhancing root regeneration, with distinct spatial expression patterns primarily observed within the adventitious root primordium and root during the second and third stages. *In situ* hybridization experiments confirmed these expression patterns, validating our spatial transcriptome findings (Fig. 5). Identifying *SAC56* and *LOS1* as marker genes offers potential applications in enhancing plant propagation techniques.

To further contextualize our findings, we compared them with the *de novo* root regeneration process in *Arabidopsis*, comprising three distinct stages—early signaling, auxin accumulation, and cell fate transition—is well-documented [12] and highly relevant to our findings. High levels of auxin signaling, crucial for defining the stem-cell organizer of the vascular cambium, were observed in cambium cells (Figs 3A and G, and 6), aligning with previous studies [6, 7, 12]. This observation is corroborated by the expression patterns of auxin-related genes in cambium cells as shown in our study. However, the manifestation might not be overt, possibly due to the delayed stage of gene expression. Approximately 12 hours post-wounding, auxin accumulation becomes evident in regeneration-competent cells, specifically in procambium and vascular parenchyma cells near the wound site in leaf explants [6].

**Figure 7:**
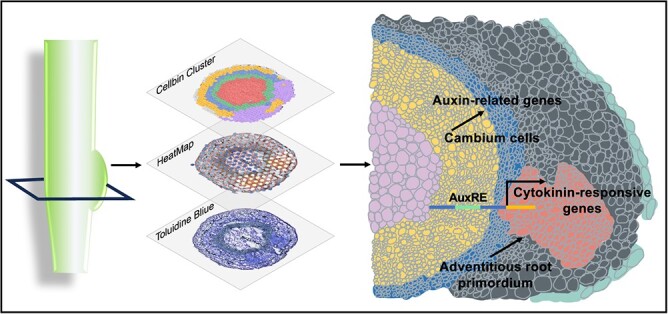
Spatial transcriptome analysis of adventitious root development. The model presents the spatial distribution of key hormone-responsive genes during root development. Auxin-related genes are predominantly expressed in the cambium region, highlighting their crucial role in root development. Cytokinin-responsive genes show elevated expression in the root primordia and meristem, which are essential for root growth. Approximately 94.17% of the promoter regions of these cytokinin-responsive genes contain AuxREs, indicating the complex genetic regulation underlying cytokinin-mediated responses in these developmental zones.

While auxin plays a significant role, cytokinin are known to promote cell division, and this activity is particularly important in the root meristem, where cells undergo rapid division. Locally synthesized cytokinin is proposed to counteract auxin concentration and restrict its distribution and auxin-cytokinin interaction guides the establishment during root regeneration [11–13, 36]. AuxREs *cis* elements have been identified in the upstream regions of target genes for auxin response factors (ARFs) [37, 38]. Previous studies have highlighted the critical role of *ARF6/8* in root development, specifically their ability to bind directly to auxin response *cis*-elements (AuxREs) on the promoters of key genes such as *RGI2* and *LBD16*. These findings have significantly enhanced our understanding of hormone interplay in root development [32]. Building on the foundational understanding of cytokinin’s role, our study builds upon this foundation by examining the expression patterns of cytokinin-responsive genes in poplar during adventitious root formation. A multitude of cytokinin-responsive genes was observed in the adventitious root primordium during the second and third stages of root development, highlighting the crucial role of cytokinin in regulating the architecture of adventitious roots. Specifically, our analysis revealed that 94.17% of cytokinin-responsive genes contain AuxREs *cis* elements in their promoters, suggesting a regulatory role for ARFs in modulating cytokinin-responsive gene expression and influencing cytokinin distribution (Figs. 3 and 7). Furthermore, we found that 94.82% cytokinin-responsive genes contain WOXCEs, indicating an overlap in the regulatory pathways governed by WOX11 and auxin. Interestingly, 89.64% of these cytokinin-responsive genes contain both AuxREs and WOXCEs in their promoters. This co-presence suggests a coordinated regulation by both auxin and cytokinin signaling pathways, underscoring the complexity of hormonal control in root morphogenesis.

In an extension of our study as reported in Song *et al.* [39], we investigated whether homologous genes of cytokinin-responsive genes in tomatoes would specifically concentrate in the bud primordium during bud regeneration. The results, presented in Fig. S4B (see online supplementary material), indicate a concentration in the outgrowth shoot. However, other hormone-responsive genes were not distinctly clustered in specific cell types, as shown in Fig. S4A, C–F (see online supplementary material).

Jasmonate plays a pivotal role in activating the auxin synthesis pathway through *ERF109*, which in turn triggers root regeneration processes [4, 5]. Our observations did not show noticeable accumulation of jasmonate response genes in root primordia or specific cell types during the second and third stages, likely due to the rapid response of jasmonate within 2 hours, leading to no discernible differential expression between these stages (Fig. 3F and L).

Abscisic acid (ABA) is a major abiotic stress-responsive hormone and plays an essential role in root growth in plants [40]. ABA promotes auxin biosynthesis and inhibits the elongation of rice primary roots [41]. Additionally, miR159a may participate in the ABA signaling pathway by inhibiting the expression of *PeMYB33*, thereby promoting the occurrence of adventitious roots [42]. ABA inhibits root cell elongation by repressing cytokinin signaling [43]. Our research results, as shown in Fig. 3B and C, reflect the spatial distribution of genes that respond to cytokinin and ABA. These genes exhibit a mutually inhibitory relationship in their spatial distribution, with cytokinin-responsive and ABA-responsive genes showing distinct, non-overlapping patterns. Additionally, investigating the interaction between other hormonal pathways could yield further insights into the regulatory networks governing root development.

The transition from adventitious root founder cells to root primordium involves critical genetic regulation by *WOX11*, leading to the activation of *WOX5/7* and *LBD16* [8, 10, 12]. In our study, the expression patterns of *WOX11/WOX12a*, *WOX5a/5b*, and *LBD16* in poplar closely resemble those observed in *Arabidopsis thaliana*, as shown in Figs 2A and F and S5 (see online supplementary material). This expression is consistent with the proposed regulatory framework, where *WOX11/12a* initiates the formation of root primordium by activating *WOX5/7* and *LBD16*. Notably, *WOX5a* has been shown to increase the number and decrease the length of adventitious roots in *Populus tomentosa* [44], which aligns with our findings of *WOX5a/5b* expression during AR development. Moreover, we have identified a role for *WOX13a* in this complex developmental pathway, detailed in Fig. S6 (see online supplementary material). To facilitate broader access to our data and support ongoing research, we have established a dedicated web server, available at http://www.lilabpku-iaas.com:8678, which serves as a comprehensive resource for additional information.

## Materials and methods

### Plant materials

We cultured the plants according to a previous paper [44] and made some modifications, as follows: Obtain the third to fourth stem segments from tissue-cultured seedlings of *Populus alba × Populus tremula var. glandulosa* that have been growing for about one month, and place them into the rooting medium (WPM + 25 g sucrose +6.5 g agar +1 mg/L VB1 + 0.05 mg/L IBA) with a 16-hour light/8-hour dark photoperiod. Each culture bottle should contain six stem segments. Collect samples daily over a week (0 d, 1 d, 2 d, 3 d, 4 d, 5 d, 6 d, 7 d), with each sample consisting of 12 stem segments, approximately 0.5 cm from the stem base. Immediately flash-freeze the samples using liquid nitrogen and store them at −80°C. Three biological replicates were performed and sequenced using the Illumina NovaSeq platform (Annoroad, Yiwu, Zhejiang Province, China).

### Bioinformatics analysis of mRNA-seq

The mRNA-seq data were aligned to the *P. alba × P. tremula var. glandulosa* (v 4.1) genome using HISAT2 [45], and eggNOG-mapper was used to functionally annotate the protein sequences [46, 47]. Transcript assembly and quantification were performed with StringTie [48]. Differentially expressed gene analysis was carried out using Trinity with the specified settings ‘—method DESeq2’ [49]. Genes were identified as differentially expressed based on fold changes greater than 2 and an adjusted *P*-value (padj) of less than 0.05.

### Spatial transcriptome library construction and sequencing

We used the MKMANU S1000 RNA-seq utilized the BMKMANU S1000 Gene Expression Kit (BMKMANU, ST03002) for our experiments. Tissue sectioning, toluidine blue staining, imaging, and initial permeabilization were conducted according to the protocols outlined in the BMKMANU S1000 Tissue Optimization Kit user manual (BMKMANU, ST03003). This was followed by a secondary permeabilization step lasting nine minutes. The cDNA library was then constructed using the BMKMANU S1000 Library Construction Kit. Sequencing was performed on the Illumina NovaSeq platform (Annoroad, Yiwu, Zhejiang Province, China).

### Promoter sequence extraction and *cis*-element analysis

TBtools [50] was used to extract the 2000 bp upstream of the coding sequence (CDS) for each gene. The extracted promoter sequences were then analysed with reference to the AuxREs and WOXCEs mentioned in published studies [32]. For statistical analysis, a promoter sequence was considered to contain AuxREs or WOXCEs if it included any form of these *cis*-elements. Specifically, if a promoter sequence contained at least one variant of an AuxRE or WOXCE, it was classified as containing AuxREs or WOXCEs.

### Analysis of spatial transcriptome data

The raw spatial transcriptomics RNA-seq data were aligned to the *P. alba × P. tremula var. glandulosa* genome [51]. Expression matrices for each gene at each location were generated using BSTMatrix v 2.2 g (http://www.bmkmanu.com/portfolio/tools). Dynamic resolution clustering of specified areas was implemented using BSTViewer (v 3.0). Further analysis was conducted using Seurat software (v 4.3.0.1) [52]. Within Seurat, normalization was carried out using SCTransform. Principal component analysis (PCA) was applied to reduce linear dimensionality. The FindClusters function in Seurat, set with a resolution of 0.5 and utilizing the first 30 principal components (PCs), identified distinct cell clusters. These clusters were visualized and examined using UMAP technique [56]. To identify genes enriched within each cluster, the FindAllMarkers function in Seurat was employed, with settings ‘min.pct = 0.25’ and ‘logfc.threshold = 0.25’.

### Pseudo-time analysis

We established a continuous developmental trajectory using the Monocle2 in R (version 2.28.0) [53, 54].

### *In* *situ* hybridization

In situ hybridization was performed according to our previous study [39]. The detailed protocol is as follows: The tissue samples were initially fixed in FAA (formalin-aceto-alcohol) fixative overnight at 4°C. After fixation, the samples were dehydrated using a gradient ethanol series and cleared with xylene. The tissues were then embedded in paraffin and sectioned at 8 μm thickness using a paraffin microtome (Thermo Scientific™ HM 355S). The cDNA fragments used as probes were amplified (refer to Table S5 (see online supplementary material) for primer details) and cloned into the pEASY-Blunt3 cloning vector. After plasmid extraction, these fragments were linearized at the vector’s restriction site. The linearized DNA was then transcribed *in vitro* and labeled with digoxigenin. The tissue sections were dewaxed with a gradient ethanol series and digested with proteinase K (Servicebio, G1234). Hybridization was performed with the labeled probes. Post-hybridization, the sections were incubated with anti-digoxigenin antibody (Jackson, 200–052-156) for 50 minutes. Signals were detected using the BCIP/NBT stock solution. Images were obtained using a microscope slide scanner (PANNORAMIC MIDI II, 3DHISTECH Ltd) in a bright field of view.

## Supplementary Material

Web_Material_uhae237

## Data Availability

The raw sequence data (spatial transcriptome data and Bulk RNA data) reported in this paper have been deposited in the CNGB Sequence Archive (CNSA) of China National GeneBank DataBase (CNGBdb) [55] with accession number CRA014595 (https://bigd.big.ac.cn/gsa/browse/CRA014595) and CRA014319 (https://bigd.big.ac.cn/gsa/browse/CRA014319).
