# Supplementary figures and images for: Spatial transcriptome analysis reveals de novo regeneration of poplar roots
Source: Hortic Res. 2024 Aug 20;11(11):uhae237. doi: 10.1093/hr/uhae237 (PMC11540759; doi:10.1093/hr/uhae237)

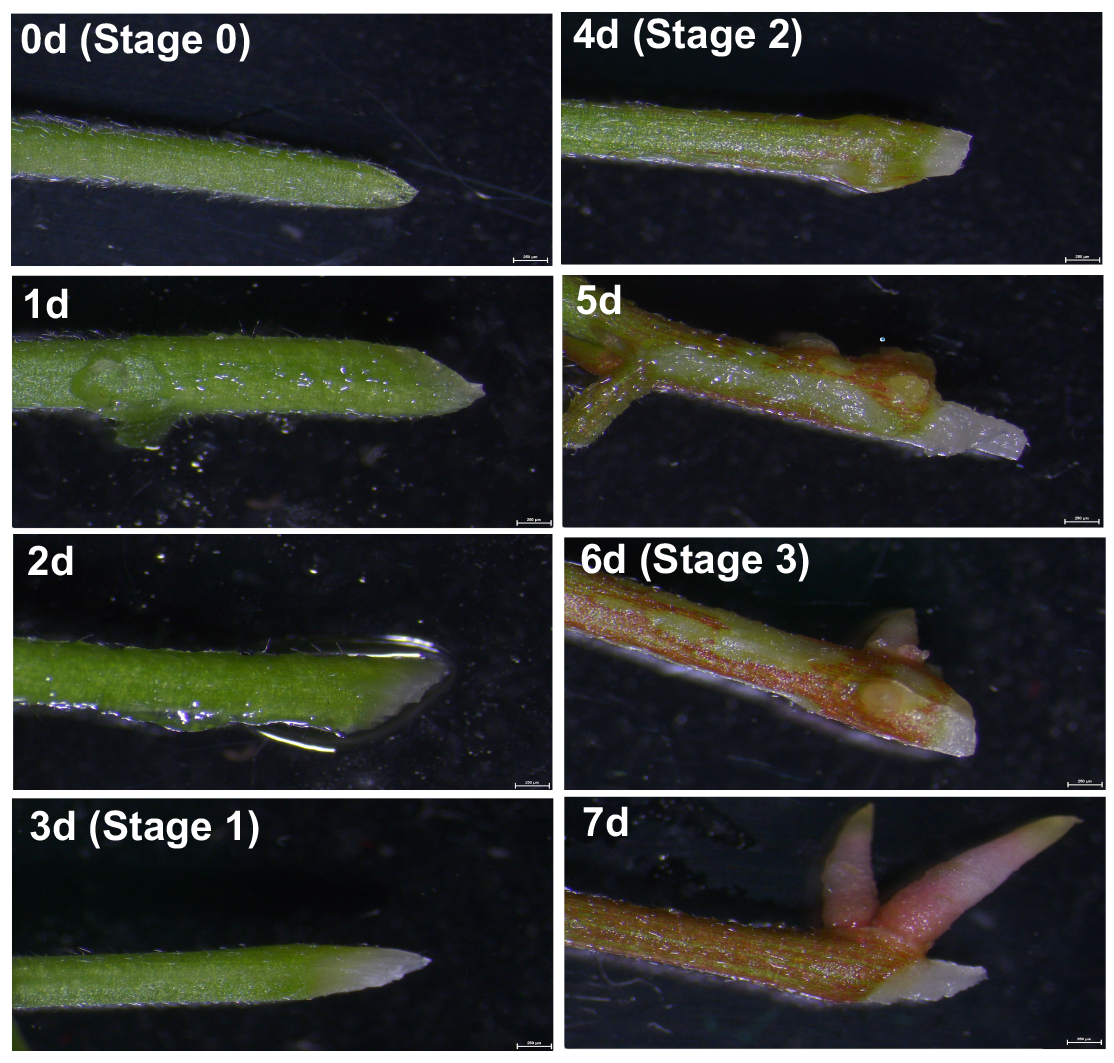

Supplement: Web_Material_uhae237 [file web_material_uhae237.zip › Supplementary Figure 1_24_06.tif]

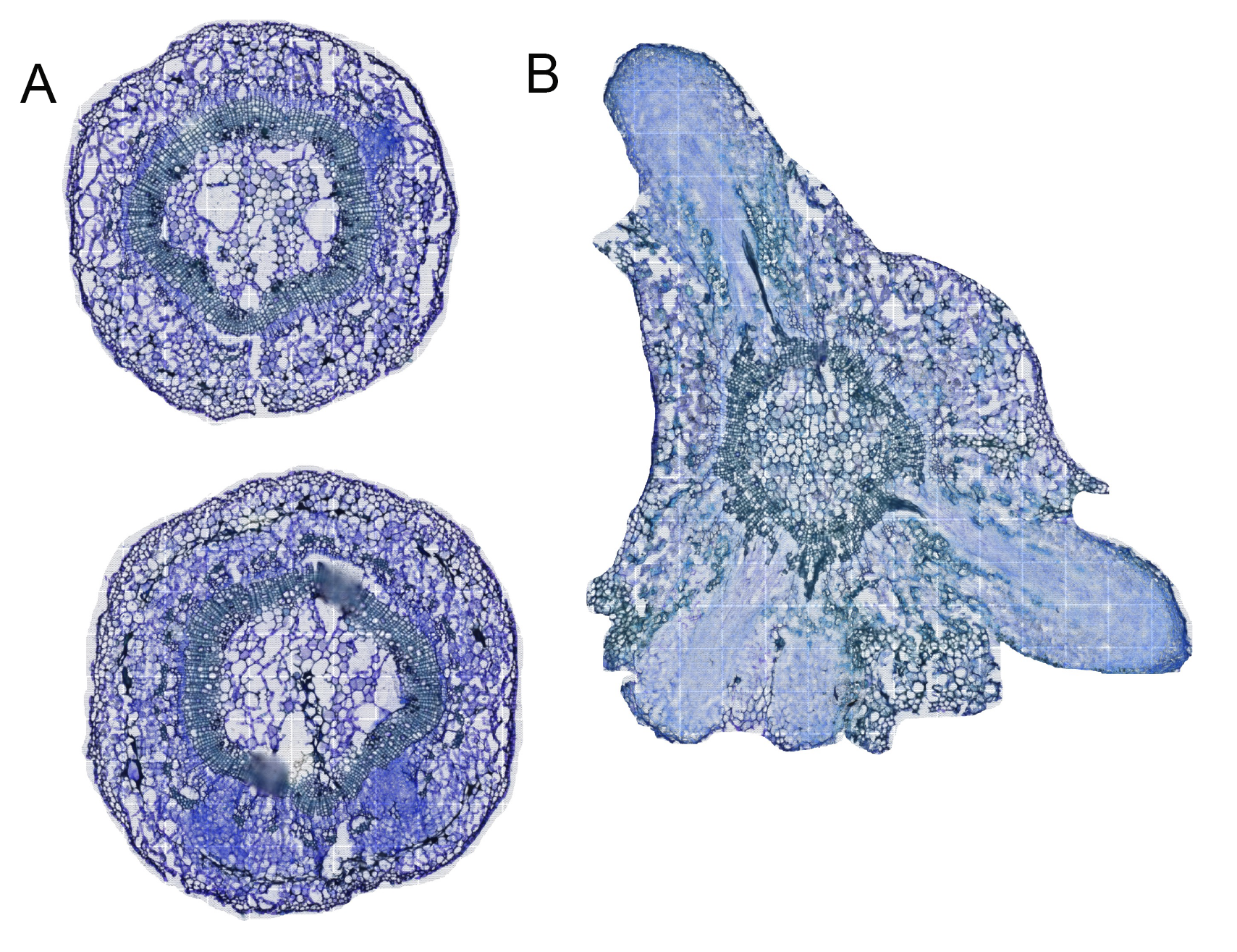

Supplement: Web_Material_uhae237 [file web_material_uhae237.zip › Supplementary Figure 2.tif]

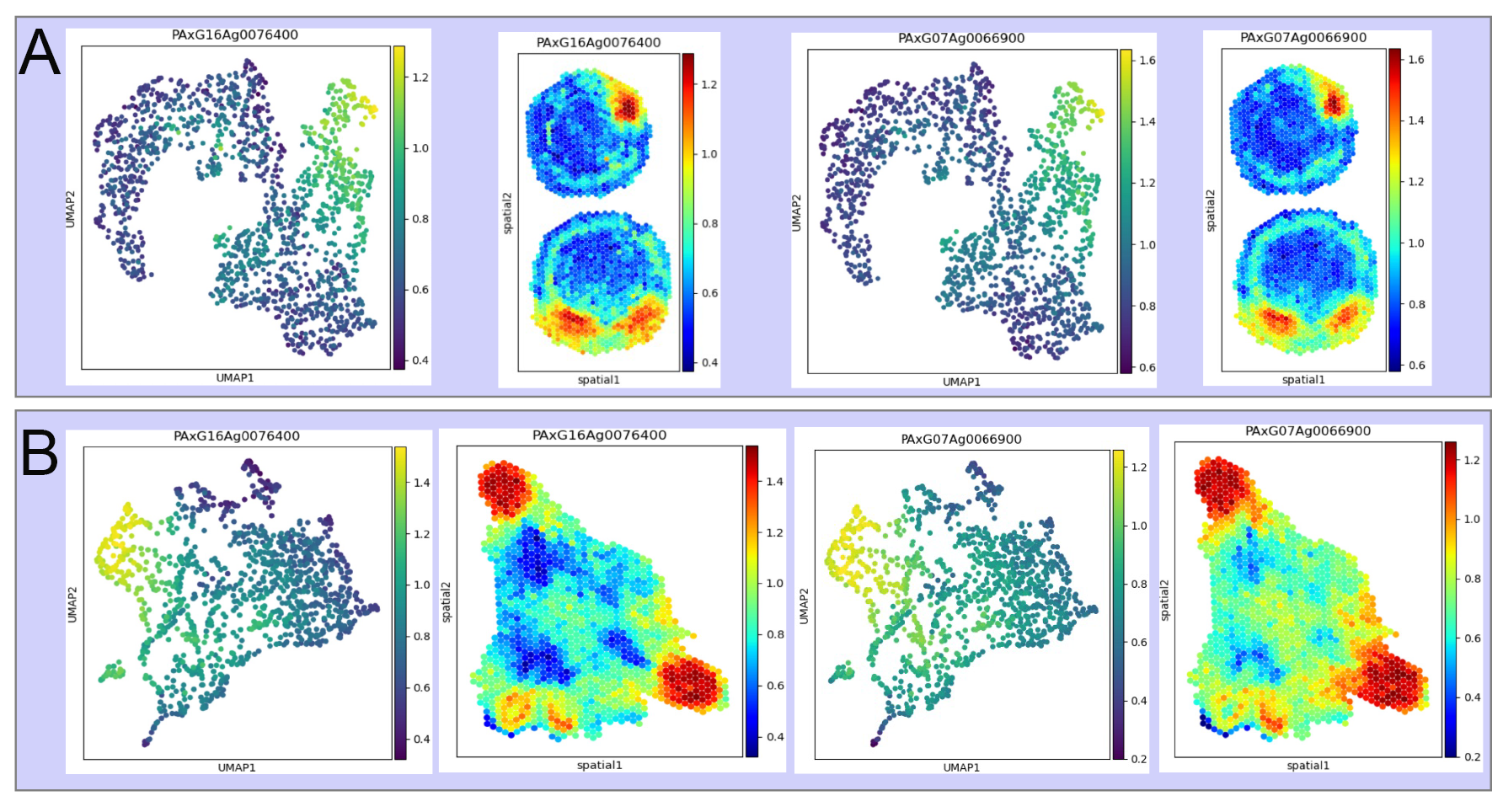

Supplement: Web_Material_uhae237 [file web_material_uhae237.zip › Supplementary Figure 3.tif]

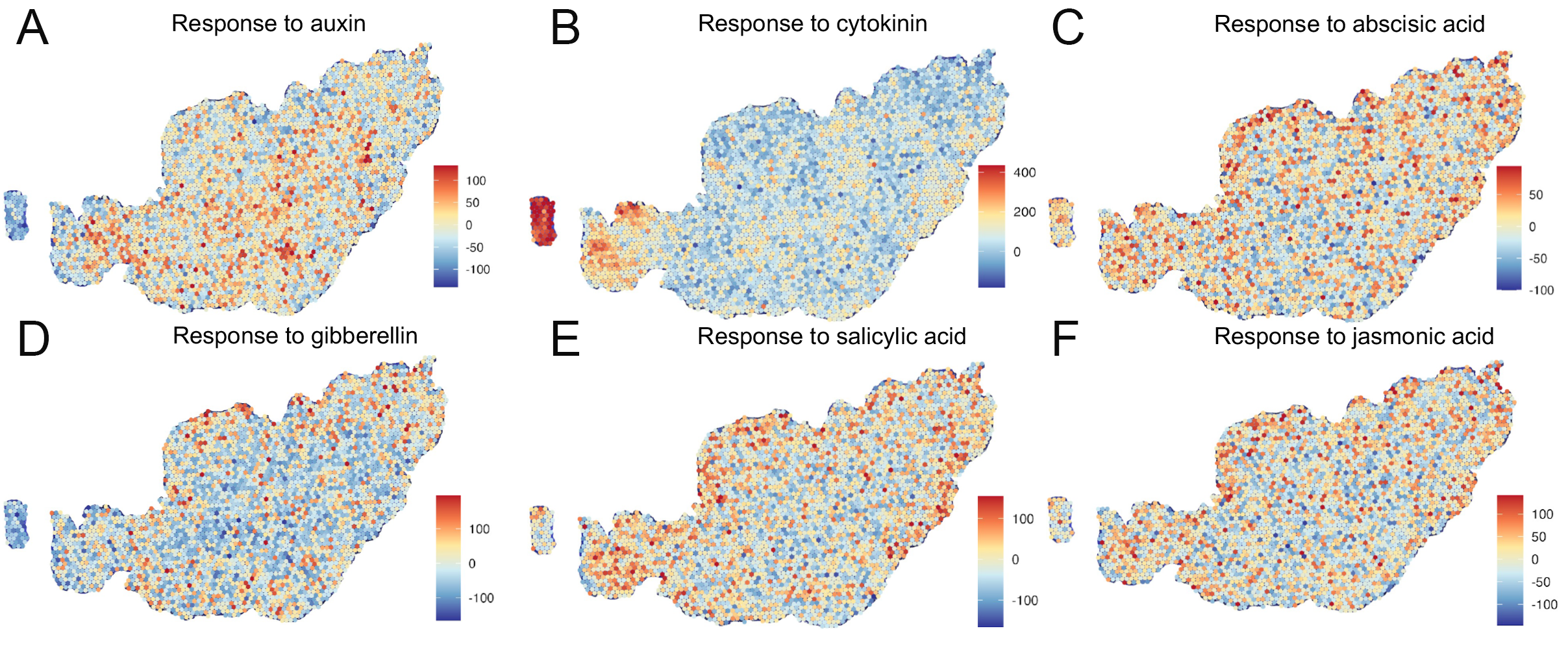

Supplement: Web_Material_uhae237 [file web_material_uhae237.zip › Supplementary Figure 4_24_06.tif]

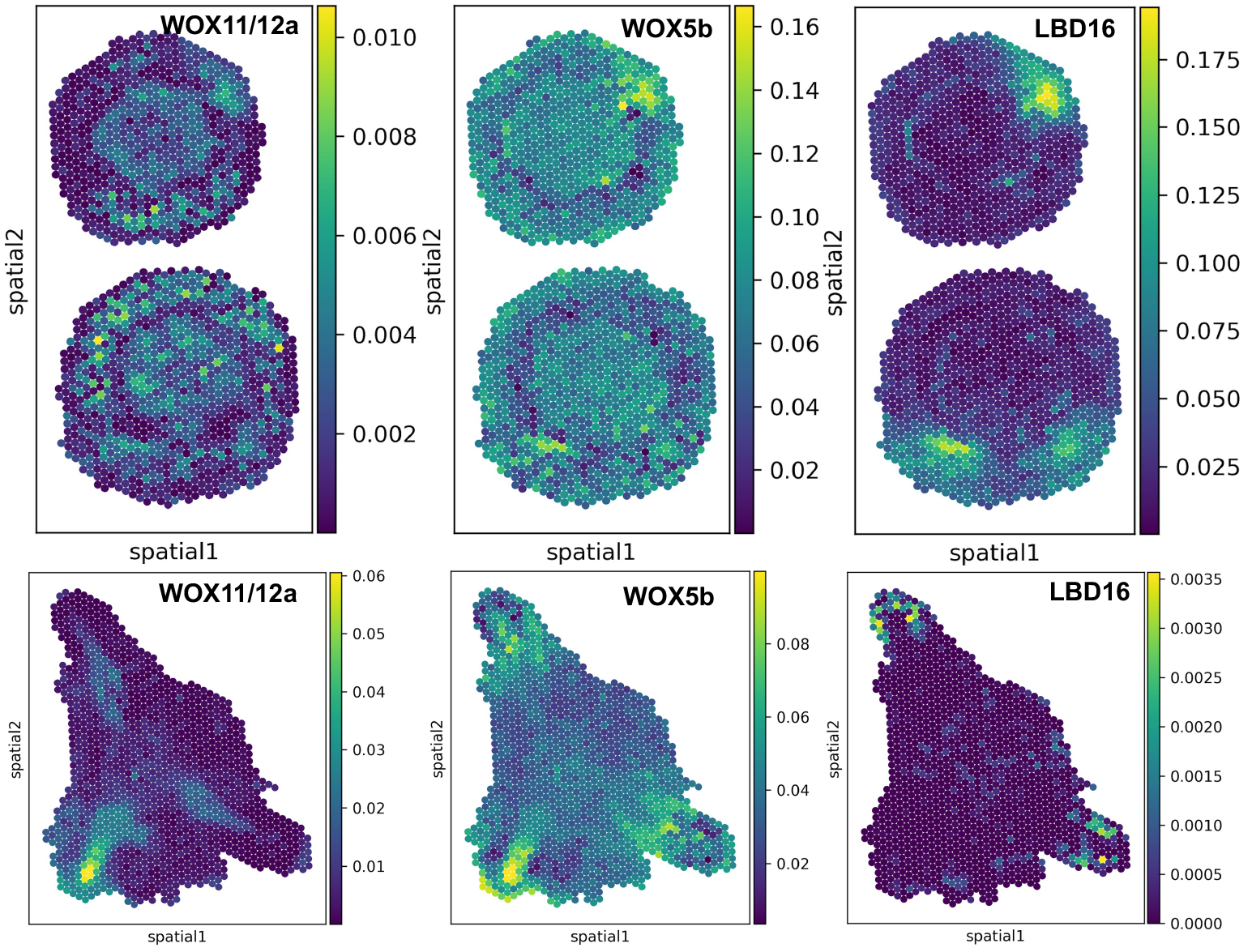

Supplement: Web_Material_uhae237 [file web_material_uhae237.zip › Supplementary Figure 5_24_06.tif]

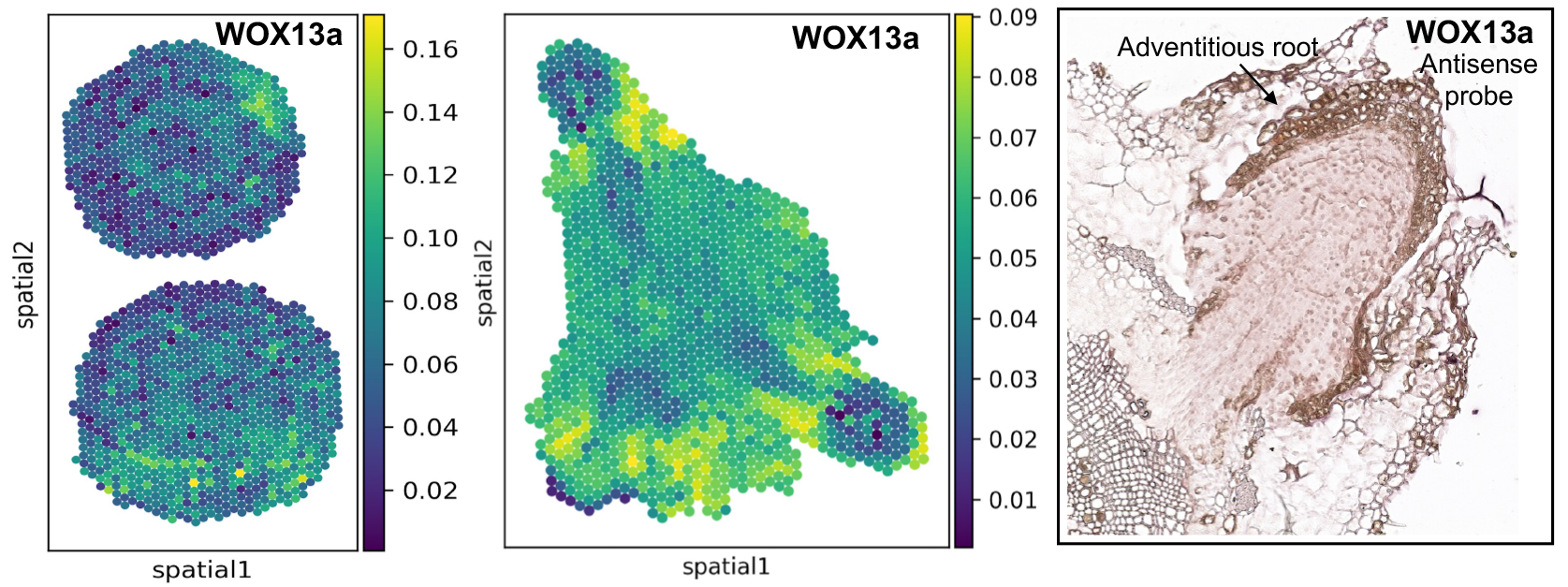

Supplement: Web_Material_uhae237 [file web_material_uhae237.zip › Supplementary Figure 6_24_06.tif]
